# A Ruptured Pseudoaneurysm of an Anomalous Gastroduodenal Artery: A Rare Presentation

**DOI:** 10.7759/cureus.14899

**Published:** 2021-05-08

**Authors:** Anish Shrestha, Anisha Shrestha, Bikal Ghimire

**Affiliations:** 1 Department of Gastrointestinal and General Surgery, Institute of Medicine, Kathmandu, NPL; 2 Department of Gastrointestinal and General Surgery, Maharajgunj Medical Campus, Institute of Medicine, Tribhuvan University, Kathmandu, NPL; 3 Department of Surgery, Institute of Medicine, Tribhuvan University Teaching Hospital, Kathmandu, NPL; 4 Department of Surgery, Tribhuvan University Teaching Hospital, Kathmandu, NPL

**Keywords:** pseudoaneurysm, gda, vaa, gastroduodenal artery, visceral artery pseudoaneurysm

## Abstract

Gastroduodenal artery (GDA) anomalies are a rare entity. Rupture of such anomalies can present with a diagnostic challenge. In this report, we describe a case of ruptured pseudoaneurysm of an anomalous GDA arising directly from the aorta presenting with recurrent abdominal pain and anemia. The diagnosis was made on computed tomography scan which showed acute retroperitoneal fluid collection. Further angiographic intervention highlighted the anomalous GDA arising directly from the aorta.

## Introduction

Celiac trunk is the artery of the foregut, arising from the abdominal aorta at the level of twelfth thoracic and the first lumbar vertebral level. It courses approximately 1.5 to 2 cm forward horizontally before dividing into three branches: left gastric, common hepatic, and splenic arteries. The gastroduodenal artery (GDA) arises as a terminal branch of the common hepatic artery along with the proper hepatic artery. A cadaveric study done by Lipshutz reported the GDA to originate from the common hepatic artery in 92.3% of the cases [1]. Another study conducted in 31 cadavers showed the GDA to originate from the common hepatic artery in 100% of the cases [2]. This emphasizes the variation in the origin of the GDA as a rare entity. There have been reports of the GDA arising from the celiac trunk or superior mesenteric artery [1,3,4], but seldom has there been any report of the GDA arising directly from the abdominal aorta. Rupture of such anomalous GDA can present with signs and symptoms different from a typical rupture.

A true aneurysm of an artery involves all the three layers of the vessel wall while a pseudoaneurysm lacks intimal and medial layers. Visceral artery aneurysm (VAA)/splanchnic artery aneurysm refers to intra-abdominal aneurysms excluding the aortoiliac axis. Some autopsy studies have suggested that splanchnic artery aneurysms occur more frequently than abdominal aortic artery aneurysms [5]; however, most of them are clinically silent [6-8]. These aneurysms are very important to recognize because the repair of a ruptured VAA has three to four-fold increased mortality and morbidity compared to an unruptured VAA [9].

Anatomical variations of visceral arteries and their aneurysms are usually diagnosed by computed tomography (CT) and/or angiography. Here, we describe the case of a pseudoaneurysm rupture presenting with abdominal mass due to retroperitoneal hematoma and abdominal pain.

## Case presentation

**Figure 1:**
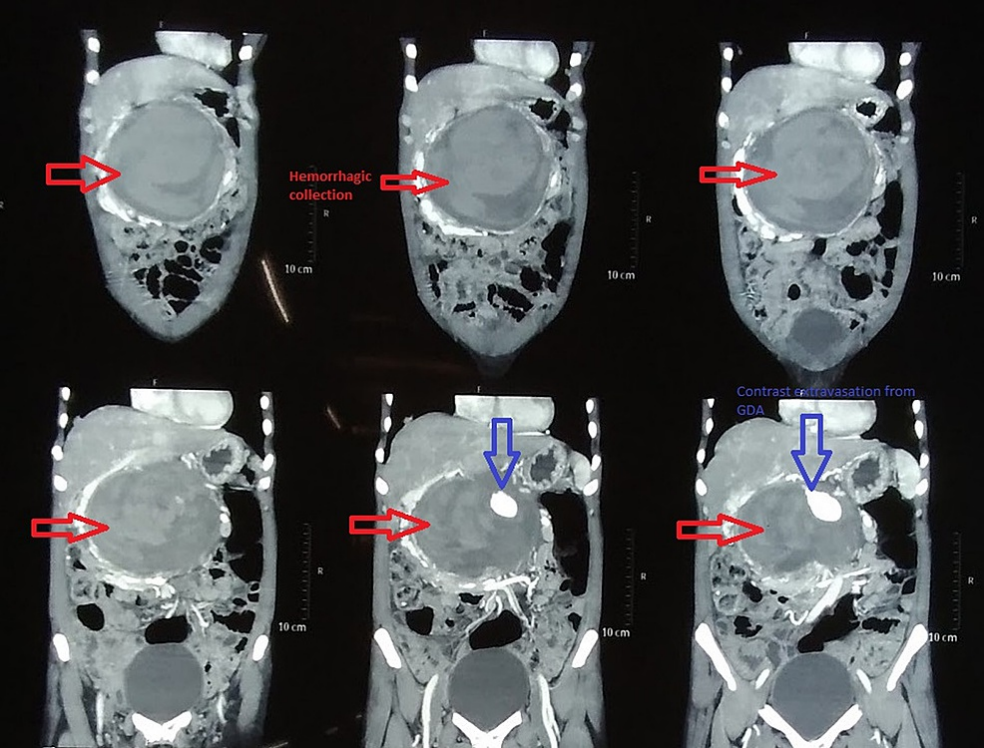
CT: Coronal sections showing retroperitoneal hemorrhagic collection. CT: computed tomography; GDA: gastroduodenal artery

**Figure 2:**
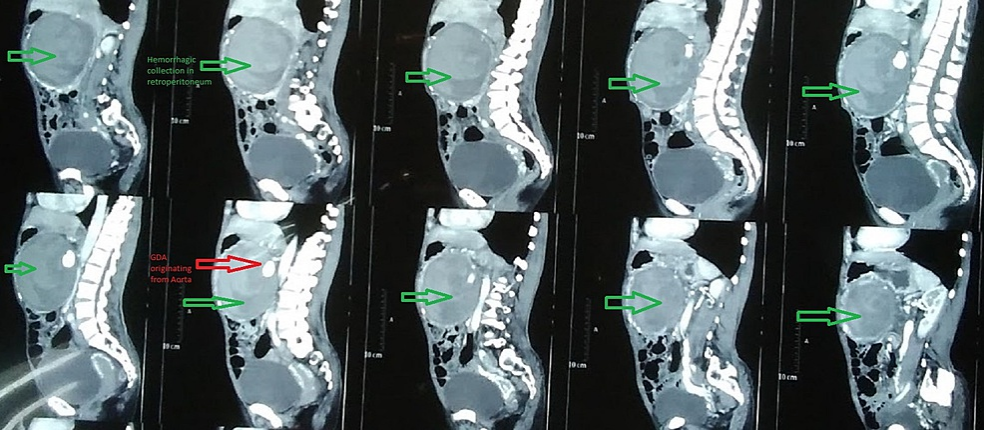
CT: Sagittal sections showing anomalous origin of the GDA from the aorta. CT: computed tomography; GDA: gastroduodenal artery

**Figure 3:**
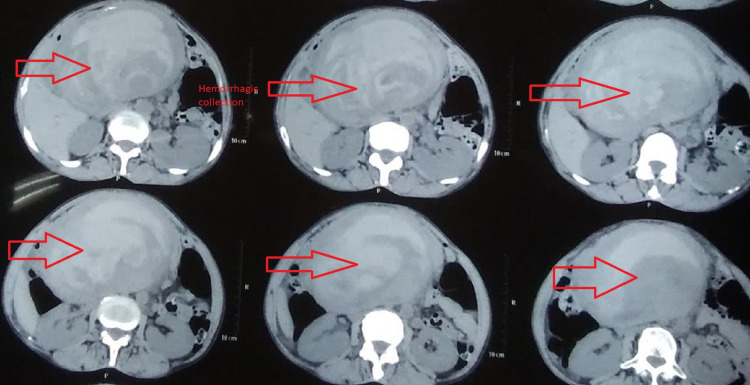
CT: Transverse sections showing retroperitoneal blood collection. CT: computed tomography

A 45-year-old female presented to the Emergency Department with a history of on and off abdominal pain for one year that evolved to continuous pain for one month along with multiple episodes of vomiting containing food particles without a history of hematemesis. She also complained of shortness of breath. On further inquiry, she gave a history of water brash on and off but denied a history of trauma or diarrhea. With a hemoglobin level of 6.4 g/dL, she had been transfused with two units of blood at a different hospital where contrast-enhanced CT scan had been performed which reported hemorrhagic collection in the upper retroperitoneal region with contrast extravasation from the GDA, with separate origin of the GDA from the aorta (Figures 1, 2, 3). Her hemoglobin at this time was 9.3 g/dL. Palpation of her abdomen revealed a 10 × 10 cm, non-pulsatile, tender mass in the upper quadrant.

The patient underwent coil embolization of the GDA via a transfemoral approach. Using a 5-Fr Cobra catheter, three coils of size 4 × 8 mm, 5 × 5 mm, and 4 × 8 mm were delivered. There was evidence of pseudoaneurysm of the GDA arising directly from the aorta with additional finding of the left hepatic artery originating from the left gastric artery. After embolization, her hemoglobin was checked every eight hours which remained static.

## Discussion

VAAs can be a true or a pseudoaneurysm. True aneurysm results from vessel wall abnormality such as atherosclerosis, cystic medial necrosis, and Marfan’s syndrome, whereas pseudoaneurysms usually result from inflammatory or traumatic conditions [5].

The incidence of VAA in autopsy ranges from 0.098% to 10.4% with an average of 1% [6,7]. The prevalence of VAA in clinical practice is reported to be 0.1% to 2% [8]. This difference signifies that most VAAs are clinically silent. About 60% of splanchnic aneurysms involve the splenic artery, 20% the hepatic artery, 5% the superior mesenteric artery, 4% the smaller branches of the celiac artery (gastric, pancreaticoduodenal, gastroepiploic), and another 4% the celiac artery [10]. Jejunal, ileal, and colic arteries represent 3% of the total number of cases [7]. The GDA accounts for 1.5% of all VAAs, most of which are pseudoaneurysms. Despite its rarity, it has a high-risk rupture of about 75% [11].

GDA pseudoaneurysm has mostly been described as a complication of chronic pancreatitis. Rarely, it has been described in association with peptic ulcer disease [12]. Our patient did not give a history suggestive of chronic pancreatitis but had a history consistent with symptoms of acid peptic disease.

Clinical presentation of GDA aneurysm rupture is varied, hence leading to a delay in the diagnosis. Gastrointestinal hemorrhage secondary to rupture of the aneurysm is the most common clinical presentation (52%), abdominal pain occurs in 46% of the cases, while 7.5% remain asymptomatic [11]. Pulsatile abdominal mass along with bruit also suggests the presence of an aneurysm, but its absence does not rule it out. GDA pseudoaneurysm can manifest with symptoms of obstructive jaundice and pancreatitis because of extrahepatic compression of the common bile duct and pancreatic duct by mass effect [13]. In addition, such a mass can cause compressive symptoms manifesting as vomiting. The same was noted in our case as well.

Although spontaneous thrombosis of pseudoaneurysm has occasionally been reported, the consecutive treatment is associated with mortality as high as 90% [14]. Endovascular intervention and open surgery are the two treatment approaches for VAAs. The choice between the two usually depends upon the hemodynamic stability of the patient as well as anatomical feasibility for endovascular repair [11,14]. In case of failure of endovascular repair, one can always resort to open surgery. In this patient, the resulting retroperitoneal hematoma from the ruptured pseudoaneurysm seemed to have had a tamponade effect preventing further extravasation of blood. It was probably due to this reason that her vitals remained stable. Therefore, we opted for endovascular repair.

Treatment of aneurysms in an emergency setting, either using an open or endovascular approach, is associated with a much higher mortality and morbidity rate than those performed in an elective setting [9]. Usually, the elective treatment is done for VAAs of size >2 cm [7]. However, its treatment should not rely completely on its size but should also depend on factors such as rate of increase in aneurysm size and location.

## Conclusions

Clinical presentations of rare entities possess a diagnostic dilemma. Early and prompt diagnosis of such conditions can save lives. We recommend contrast-enhanced CT abdomen scans as a diagnostic aid in such situations.
